# Autophagy induction halts axonal degeneration in a mouse model of X-adrenoleukodystrophy

**DOI:** 10.1007/s00401-014-1378-8

**Published:** 2014-12-31

**Authors:** Nathalie Launay, Carmen Aguado, Stéphane Fourcade, Montserrat Ruiz, Laia Grau, Jordi Riera, Cristina Guilera, Marisa Giròs, Isidre Ferrer, Erwin Knecht, Aurora Pujol

**Affiliations:** 1 Neurometabolic Diseases Laboratory, Bellvitge Biomedical Research Institute (IDIBELL), L’ Hospitalet de Llobregat, 08908 Barcelona, Catalonia Spain; 2 Institut of Neuropathology, Pathologic Anatomy Service, Bellvitge Biomedical Research Institute, IDIBELL-Hospital Universitari de Bellvitge, L’Hospitalet de Llobregat, 08908 Barcelona, Catalonia Spain; 3 Center for Biomedical Research on Rare Diseases (CIBERER), ISCIII, Madrid, Spain; 4 Laboratory of Cellular Biology, Centro de Investigación Príncipe Felipe, 46012 Valencia, Spain; 5 Institut de Bioquímica Clínica, Corporació Sanitària Clinic, 08028 Barcelona, Catalonia Spain; 6 Center for Biomedical Research on Neurodegenerative Diseases (CIBERNED), ISCIII, Madrid, Spain; 7 Catalan Institution of Research and Advanced Studies (ICREA), 08010 Barcelona, Catalonia Spain

**Keywords:** X-ALD, Autophagy, mTOR, Temsirolimus, VLCFA

## Abstract

X-linked adrenoleukodystrophy (X-ALD) is a rare neurometabolic disease characterized by the accumulation of very long chain fatty acids (VLCFAs) due to a loss of function of the peroxisomal transporter ABCD1. Here, using in vivo and in vitro models, we demonstrate that autophagic flux was impaired due to elevated mammalian target of rapamycin (mTOR) signaling, which contributed to X-ALD pathogenesis. We also show that excess VLCFAs downregulated autophagy in human fibroblasts. Furthermore, mTOR inhibition by a rapamycin derivative (temsirolimus) restored autophagic flux and inhibited the axonal degenerative process as well as the associated locomotor impairment in the *Abcd1* ^−^ */Abcd2* ^−*/*−^ mouse model. This process was mediated through the restoration of proteasome function and redox as well as metabolic homeostasis. These findings provide the first evidence that links impaired autophagy to X-ALD, which may yield a therapy based on autophagy activators for adrenomyeloneuropathy patients.

**Electronic supplementary material:**

The online version of this article (doi:10.1007/s00401-014-1378-8) contains supplementary material, which is available to authorized users.

## Introduction

In addition to the ubiquitin–proteasome pathway, macroautophagy (autophagy, herein) is the major cellular process responsible for protein turnover in eukaryotic cells [30, 37, 55]. Autophagy is initiated by a double-membrane structure that closes to form an autophagosome, which contains cytoplasmic material, including organelles, protein aggregates and lipids. Next, the autophagosome fuses with endosomes/lysosomes to form a single-membrane autolysosome that degrades the sequestered material [42, 53, 59, 77].

Autophagy occurs at low basal levels in almost all cells, but it increases rapidly and strongly under certain conditions such as starvation, high energy demands [42, 53], oxidative stress and protein aggregate accumulation [28, 75].

A growing body of evidence indicates that constitutive autophagy is indispensable for maintaining neural tissue homeostasis and normal function. Dysfunctional autophagy has been observed during neurodegeneration in both in vivo animal models [10, 33, 62, 86, 87, 92] and in vitro primary neuronal cultures [6, 38, 70]. Insufficient autophagy is characterized by the accumulation of autophagic structures and has been observed in the brains of patients with neurodegenerative diseases, particularly those associated with protein aggregates such as Alzheimer’s (AD), Parkinson’s and Huntington’s diseases (HD) [58].

In this study, following previous observations of poly-ubiquitinated protein accumulation in X-linked adrenoleukodystrophy models [39] (X-ALD: McKusick no. 300100), we investigated whether autophagy is altered in X-ALD and the extent to which this impairment contributes to the axonal degeneration pathophysiology observed in this disease. X-ALD is a severe and often lethal inherited neurometabolic disorder characterized by progressive demyelination in the central nervous system, axonopathy in the spinal cord and adrenal insufficiency [13, 15, 57]. X-ALD results from a loss of function by the peroxisomal ABCD1 fatty acid transporter, resulting in VLCFAs accumulation in the target organs and plasma caused by impaired VLCFAs import into the peroxisome, which decreases the peroxisomal beta-oxidation enzyme substrate [19, 83, 85, 88]. Three major disease variants have been described. One variant is a late-onset form that affects adults and is referred to as adrenomyeloneuropathy (AMN), which is characterized by peripheral neuropathy and distal axonopathy in corticospinal tracts of spinal cords undergoing spastic paraparesis as a major symptom without brain inflammatory demyelination. Two ultimately lethal forms include cerebral demyelination and neuroinflammation; one is an adult form referred to as cerebral adrenomyeloneuropathy (cAMN), and the other is a childhood form referred to as cerebral childhood adrenoleukodystrophy (cCALD). All clinical phenotypes can occur within the same family; thus, the phenotype and genotype do not correlate [13, 15, 57]. In recent years, we and others have shown that excess VLCFAs generate reactive oxygen species (ROS) and this together with energetic imbalances underlie disease pathogenesis [18, 22, 63, 76]. However, the method by which excess VLCFAs result in adrenal and spinal cord pathologies remains elusive, including whether or not they trigger central demyelination. Therapeutic advances include allogeneic bone marrow transplant [2] and gene therapy [9]; although, these therapies can only be applied to a subset of patients upon demyelination onset within a narrow window of opportunity. A satisfactory pharmacological treatment has not yet been identified [4].

Here, we used biochemical, morphological and functional assays to directly analyze the autophagic system status in X-ALD fibroblasts, patient brains and X-ALD mouse models (*Abcd1* ^−^ and *Abcd1* ^−^ */Abcd2* ^−*/*−^). We report aberrant mTOR signaling that causes autophagy impairment as a mechanistic and pivotal component of X-ALD pathogenesis. Based on preclinical test results from a mouse model using the rapamycin analog temsirolimus, we propose using rapamycin-related mTOR inhibitors as a potential therapeutic approach for X-ALD.

## Materials and methods

### Antibodies and reagents

The following antibodies were used for Western blots: γ-tubulin and DNP (2,4-dinitrophenylhydrazone) (Sigma); LC3 (Nanotools), phospho-p70S6 kinase (Thr389), phospho-ULK1 (Ser757), p70S6 kinase and ULK1 (Cell Signaling Technology); polyUbiquitin (Dako) and K48-linked polyUbiquitin (Cell Signaling); amyloid precursor protein (Boehringer); p62 and 8-oxodG (Abcam); Iba-1 (Wako); and GFAP, synaptophysin, goat anti-rabbit and anti-mouse IgG linked to horseradish peroxidase (DakoCytomation). The fluorogenic peptides Suc-Leu-Leu-Val-Tyr-7-amido-4-methylcoumarin (Suc-LLVT-AMC) and 7-amino-4-methylcoumarin (AMC) were obtained from Calbiochem. Hexacosanoic acid (C26:0) and bafilomycin A1 were purchased from Sigma.

### Human brain samples

Brain tissue (frontal lobe unaffected white matter for patients and controls, plus affected white matter of parietal and occipital lobes) was obtained from patients with cerebral adrenoleukodystrophy (cCALD), cerebral adrenomyeloneuropathy (cAMN) and healthy age-matched male control subjects from the Brain and Tissue Bank for Developmental Disorders at the University of Maryland, Baltimore [74] (Supplemental Table 1). There was no available tissue from pure AMN patients in this Bank, which prevented us from comparisons to this phenotype. Informed written consent was obtained from all patients or their legal representatives; the local ethics committee approved the studies.

### Mouse colonies

The methods in this study were employed in accordance with the Guide for the Care and Use of Laboratory Animals published by the US National Institutes of Health (NIH publication No. 85-23, revised 1996) as well as the ethical committees of IDIBELL and the Generalitat de Catalunya (DAAM 3546). The generation and genotyping of *Abcd1* ^−^ (Abcd1^Tm1Kds^) and *Abcd2* ^−/−^ (Abcd2^Tm1Apuj^) mice have been previously described [16, 46, 64, 65]. The *Abcd1* ^−^ mice in a 129sv background were a kind gift of Dr Kirby Smith (KKI, Baltimore, MD) [46]. The *Abcd1* ^-^ and *Abcd1* ^−^/*Abcd2* ^−/−^ mice were kept at 22 °C with equal periods of darkness and light and had free access to water and food. All mice used for experiments were on a pure C57BL/6 J background. Animals were killed and tissues were recovered and conserved at −80 °C.

### Treatments and locomotor experiments

Two X-ALD mouse models were used in this study. The first model was *Abcd1* ^−^ mice that exhibit biochemical signs of pathology, including oxidative stress [17] and altered energy homeostasis [23]; however, the first clinical signs of AMN (axonopathy and locomotor impairment) appear at 20 months [64, 65]. We characterized the biochemical signs of adult AMN in these mice. The second model was mice with a double gene knockout of both the *Abcd1* and *Abcd2* transporters (*Abcd1* ^−^ */Abcd2* ^−*/*−^). Compared with the *Abcd1* ^−^ mice, the *Abcd1* ^−^ */Abcd2* ^−*/*−^ mice display enhanced VLCFAs accumulation in the spinal cord [64], higher levels of oxidative damage to proteins [20], and a more severe AMN-like pathology with an earlier onset at 12 months of age [16, 64, 65]; therefore, this is the preferred model for assaying therapeutic strategies. Notably, no disease-causative role for *ABCD2* has been demonstrated; however, its absence induces a partially overlapping fatty acid pattern compared with the *Abcd1* ^−^ dependent biochemical phenotype [19, 43]. We assessed the clinical signs of AMN in these double *Abcd1* ^−^ */Abcd2* ^−*/*−^ mice. The mice used for the experiments herein were produced using a pure C57BL/6 J background. Generation and genotyping for *Abcd1* ^−^ and *Abcd1* ^−^ */Abcd2* ^−*/*−^ mice have been previously described [46, 64]. For the temsirolimus treatments, the *Abcd1* ^−^, *Abcd1* ^−^ */Abcd2* ^−*/*−^ and wild-type littermate mice were separated into control and treated groups. Temsirolimus (LC Laboratories) was administered through intraperitoneal injections of 20 mg/kg three times a week [52, 67]. We first tested different dosages to minimize unwanted effects. Continuous temsirolimus administration over 3 months at 20 mg/kg produced substantial weight loss, between 15 and 20 % of the body weight (data not shown), which is consistent with previous observations in rodents [84]. Thus, we alternated 3-week rest periods between each month of the 3 months of treatment to a total period of 4.5 months, with mice starting treatment at 12 months and being killed after locomotor tests at 17 months of age. Under these conditions, no significant weight loss was observed, indicating that the protocol with resting periods was more suitable. We provided the control mice with a mock injection that contained buffer at the same frequency. The treadmill experiment was performed exactly as previously described [44, 56]. We measured the latency to falling off the belt (shock time) and number of shocks received. The bar cross experiment was performed as previously described [16, 44, 56]. Mock- and drug-treated mice in each litter were simultaneously tested using the treadmill and bar cross for both genotype and treatment in a blind study.

### Cell culture and treatment

Skin biopsies were used to prepare fibroblasts and were collected in accordance with the institutional guidelines for sampling, including informed consent from the persons involved or their representatives (12/062). Fibroblasts were obtained from pure AMN patients and healthy age-matched male control subjects with an age ranging from 31 to 52 years. Primary human fibroblasts were cultured in DMEM (containing 10 % fetal bovine serum, 100 U/ml penicillin and 100 µg streptomycin) at 37 °C in humidified, 5 % CO_2_/95 % air. Unless otherwise stated, the experiments were performed with cells at 80 % confluence. Control and X-ALD human fibroblasts were treated in medium containing fetal bovine serum (10 %) for 24 h with ethanol as control or C26:0 (50 μM). For starvation conditions, after washing, the cells were changed from a full growth medium (low proteolysis medium, L) to Krebs–Henseleit medium (118.4 mM NaCl, 4.75 mM KCl, 1.19 mM KH_2_PO_4_, 2.54 mM MgSO_4_, 2.44 mM CaCl_2_·2H_2_O, 28.6 mM NaHCO_3_, and 10 mM glucose) containing 10 mM HEPES, pH 7.4 (high proteolysis medium, H) and incubated for 4 h or less at 37 °C.

### Autophagy analysis in cultured cells

For pulse-chase experiments, human control and X-ALD patients’ fibroblasts were incubated for 48 h in fresh full medium with 1 µCi/ml [^3^H]valine (Hartmann Analytic GmbH) followed by a 24 h chase in fresh full medium containing 10 mM l-valine to degrade short-lived proteins [21]. Next, all cultures were incubated for the indicated times in Krebs–Henseleit medium with 10 mM HEPES, pH 7.4, 10 mM valine and the additional components indicated. Protein degradation was analyzed 1 h thereafter to ensure the maximum effects of the various added components and for only 3 h to avoid possible secondary effects. We calculated protein degradation at two 1.5-h intervals by measuring the net release of trichloroacetic acid-soluble radioactivity from the labeled cells into the culture medium, which is expressed as a percentage of the protein degraded. The contribution of lysosomal degradation to total protein degradation was calculated using 20 mM NH_4_Cl (Sigma-Aldrich) and 100 µM leupeptin (Peptide Institute), as previously described [21].

Autophagic flux was assessed by measuring endogenous LC3-II levels relative to the γ-tubulin levels after 4 h in the presence of 400 nM bafilomycin A1 using specific antibodies. This assay was previously established using various autophagy modulators [31, 69, 72]. Autophagy was also assessed by transfecting human fibroblasts with a pEGFP-LC3 (a generous gift of Noboru Mizushima, Tokyo Medical and Dental University) and after 48 h, the cells were incubated in high proteolysis medium for 2 h and the number of fluorescent dots per transfected cells were counted using a fluorescence microscope as previously described [1]. In addition, to assess autophagosome maturation, human fibroblasts were also transfected for 24 h with mRFP-GFP-LC3 (Addgene) and incubated as above. Fluorescence preparations with EGFP-LC3 and RFP-GFP-LC3 were observed and images acquired as previously described [24].

### Reverse transcription (RT)-PCR analysis

Total RNA was isolated from homogenized spinal cords using the RNeasy Mini Kit (Qiagen) in accordance with the manufacturer’s instructions. Next, the first-strand cDNA was synthesized for each RNA sample using Superscript II reverse transcriptase (Invitrogen) and oligo-dT. Expression levels of the candidate proteasome genes were analyzed through RT-PCR using TaqMan^®^ Gene Expression Assays (Applied Biosystems). The expression levels were relatively quantified using the ‘Delta–Delta Ct’ (ΔΔCt) method with RPL0 as an endogenous control. Transcript quantification was performed in duplicate for each sample [39].

### Immunohistochemistry

Spinal cords were harvested from 20-month-old wild-type, *Abcd1* ^−^ */Abcd2* ^−*/*−^ and *Abcd1* ^−^ */Abcd2* ^−*/*−^ mice treated with temsirolimus after perfusion using 4 % paraformaldehyde (PFA) as previously described [16, 44, 56, 64]. The spinal cords were embedded in paraffin, and serial sections (5 μm thick) were cut in a transversal or longitudinal (1-cm-long) plane. The number of abnormal specific profiles was counted every 10 sections for each stain. At least three sections of the spinal cord were analyzed per animal and per stain. The sections were stained with Sudan black or processed through immunohistochemistry for GFAP, Iba-1, APP, synaptophysin, and 8-oxodG. The number of abnormal specific profiles was quantified, and the results are expressed as the mean ± standard deviation.

### Electron microscopy

L1–L2 sections of mouse spinal cords were perfused with 3 % glutaraldehyde/4 % PFA in phosphate buffer (100 mM phosphate buffer, pH 7.4) at 4 °C for 24 h. Vibratome Sections (50–100-μm thick) were post-fixed in 1 % osmium tetroxide, suspended in 2 % (w/v) aqueous uranyl acetate for 1 h, washed three times in distilled water, dehydrated through a graded acetone series at 30, 50 70, 90 and 100 % and embedded in Durcupan ACM (Electron Microscopy Sciences) using standard procedures. To select the area of interest, semi-thin sections (1.5 µm) were first obtained using a diamond knife and stained with 1 % toluidine blue. Ultrathin sections were then cut, stained with Reynold’s lead citrate and observed using a Philips CM-10 electron microscope at 60 kV. Lysosomal dense bodies were identified by morphological criteria previously described [32] and counts were performed for each genotype using more than thirty electron micrographs (final magnification, 7,000×) collected at random from four different mice.

### Electrophoresis and Western blotting

Tissues were removed from euthanized mice and flash-frozen using liquid nitrogen. The frozen tissue and human fibroblasts samples were homogenized in RIPA buffer (150 mM NaCl, 1 % Nonidet P40, 0.5 % sodium deoxycholate, 0.1 % SDS, 50 mM Tris, pH 8.0) containing 0.1 mM leupeptin and 1 mM PMSF) using a motor-driven grinder (Sigma-Aldrich) and then sonicated for 2 min at 4 °C in an Ultrasonic processor UP50H (Hielscher-Ultrasound Technology). We used polyacrylamide (10 % acrylamide) gel electrophoresis for 60 min at 120 V to analyze the samples. The resolved proteins were transferred onto nitrocellulose membranes and the proteins were detected using an ECL Western blotting analysis system followed by exposure to CL-XPosure Film (Thermo Scientific). Autoradiographs were scanned and quantified using a GS800 Densitometer (Bio-Rad).

To detect protein carbonyls, the proteins were transferred to nitrocellulose membranes and derivatized with DNPH as previously described [68]. After derivatization, the membranes were blocked with 5 % free fatty acid milk and incubated with a monoclonal anti-DNP antibody (dilution: 1/1,000, ref D8406, Sigma-Aldrich) for 36 h at room temperature. Goat anti-mouse IgG linked to horseradish peroxidase (dilution: 1/10,000, ref 2015-08, Dako, Denmark) was used as a secondary antibody. The protein was detected as described above.

### ATP levels and chymotrypsin-like activity

ATP levels were quantified as previously described [23]. For proteasome activity assays, the tissue was homogenized in an ice-cold buffer (50 mM Tris–HCl pH 7.5, 1 mM dithiothreitol, 0.25 M sucrose, 5 mM MgCl2, 0.5 mM EDTA and 2 mM ATP) using a Teflon-on-glass homogenizer and centrifuged at 12,000×*g* for 10 min. Chymotrypsin-like activity was determined as described [39] using Suc-LLVY-AMC as substrate. Equal extract levels were incubated with the substrate (100 µM) in 100 µl of proteasome activity assay buffer (0.5 mM Tris–HCl, pH 7.8, 10 mM MgCl_2_ and 1 mM dithiothreitol with or without 5 mM ATP) for 30 min at 37 °C. The reactions were quenched by adding 0.9 ml of cold ethanol. The free AMC fluorescence was quantified using a fluorescence multi-plate FLUOstar OPTIMA FL reader (BMG) with excitation and emission wavelengths at 380 and 460 nm, respectively. Lactacystin (5 µM, 2 h) was employed to ensure assay specificity. All reactions were performed in duplicate and the readings were calibrated using standard fluorophore solutions.

### Statistical analyses

Statistical significance was assessed using Student’s *t* test when two groups were compared. In analyzing multiple groups, we used ANOVA and Tukey’s hsd post hoc test to determine the significance. The data are presented as the mean ± SD, and *p* < 0.05 was considered significant (*p < 0.05; **p < 0.01; ***p < 0.001).

## Results

### Autophagy is impaired in X-ALD patients and mouse models

**Fig. 1:**
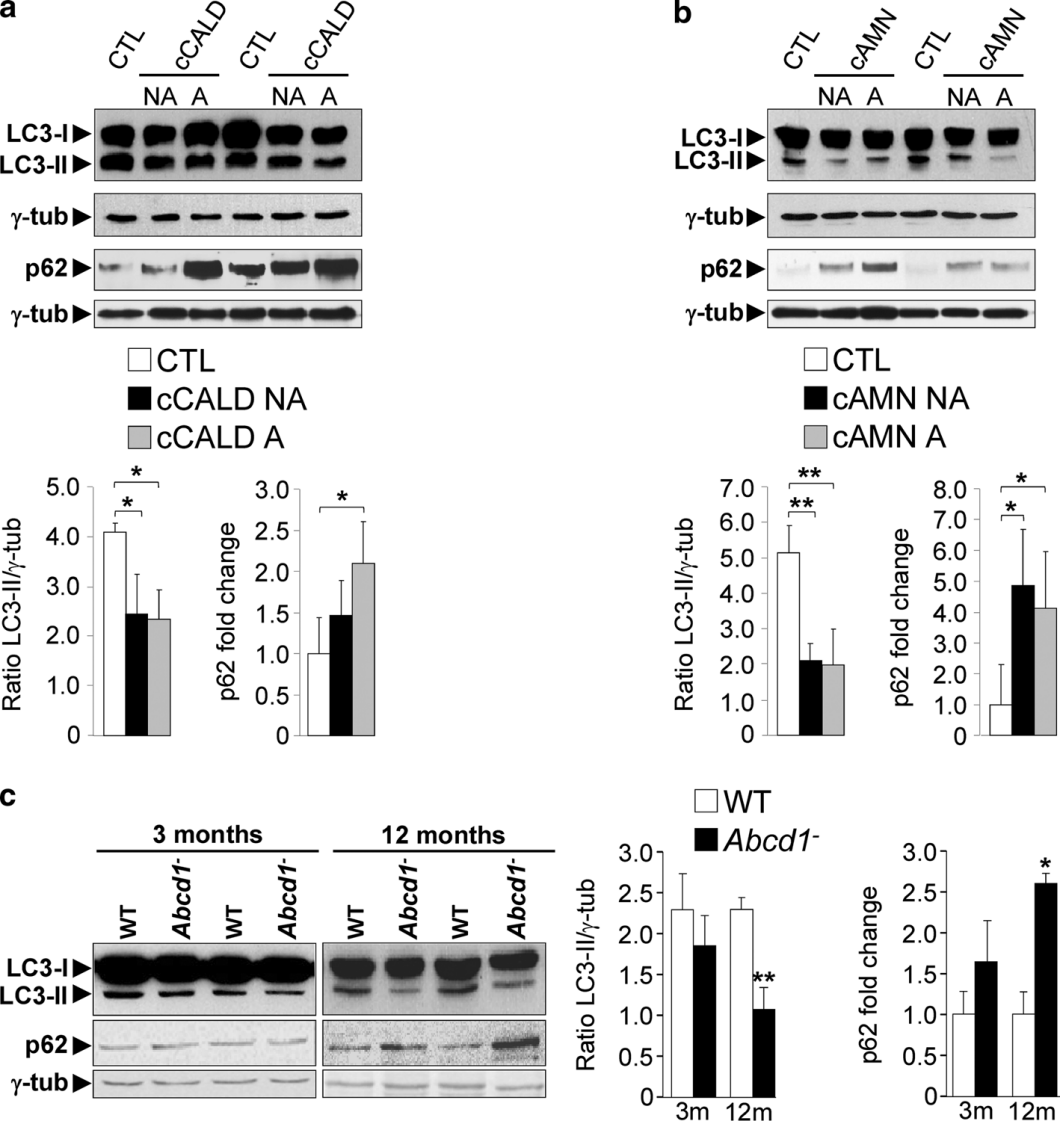
Autophagy is downregulated in brain areas of X-ALD patients and in spinal cords of *Abcd1* ^−^ mice. **a**, **b** Representative immunoblots for p62 and LC3-II in control (CTL) and in non-affected (NA) and affected (A) white matter from cCALD (**a**) and cAMN patients (**b**). **c** Representative immunoblots for p62 and LC3-II in spinal cord from WT and *Abcd*1^−^ mice at 3 and 12 months (m) of age. Protein levels are normalized respect to γ-tubulin (γ-tub). The histograms *below* (**a** and **b**) and on the *right* (**c**) show the LC3-II levels and the p62 levels relative to CTL. Values are expressed as mean ± SD (*n* = 4 samples by condition in **a** and **b**; *n* = 6 samples by genotype and age in **c**; *p < 0.05 and **p < 0.01, one-way ANOVA followed by Tukey’s hsd post hoc test for **a** and **b**, Student’s *t* test for **c**)

We examined the expression patterns of two molecular autophagy indicators in patients who suffered from the cerebral forms of X-ALD: cCALD and cAMN. First, we used the classical autophagosome marker LC3. During macroautophagy activation, the cytosolic protein LC3-I is converted into LC3-II by lipidation, and it specifically associates with both sides of the limiting membranes that form the autophagosome. LC3-II does not bind other organelles and is degraded in lysosomes in an autophagy-dependent manner. We assessed the LC3-II levels in the unaffected and affected brain areas of cCALD and cAMN patients as well as in control samples (Fig. 1a, b) and found that they were consistently lower in both the patients’ unaffected and affected brain areas. Because LC3-II levels depend on the rates of both autophagosome formation and conversion into autolysosomes (in the absence of lysosomal inhibitors), this decrease indicates either impaired autophagy or increased autophagosome–endosome/lysosome fusion. Therefore, we measured the levels of the multifunctional protein p62 (also referred to as SQSTM1), which is a protein involved in aggresome formation that can be degraded by autophagy [5]. We found that p62 levels were elevated in unaffected areas and, more prominently, in the affected zones of both cCALD and cAMN patients (Fig. 1a, b). These results support the notion that autophagy is impaired in X-ALD patients.

Next, we studied both autophagy markers in an X-ALD mouse model at different stages of the disease. The X-ALD mouse model is a classic *Abcd1* gene knockout model (*Abcd1* ^−^ mice), which exhibits late-onset axonopathy in the spinal cord without overt inflammation or demyelination; thus, it resembles adult onset adrenomyeloneuropathy in humans [65]. *Abcd1* ^−^ mice present overt locomotor disabilities and axonopathy at 20–22 months of age; however, oxidative damage appears very early, at approximately 3 months of age [17]. We also observed impaired autophagy in 12-month-old *Abcd1* ^−^ mouse spinal cords, as evidenced by lower LC3-II levels and elevated p62 levels compared with wild-type mice (Fig. 1c). These differences were not significant in 3-month-old *Abcd1* ^−^ mice compared with wild-type mice (Fig. 1c), which indicates an accumulative phenotype over time.

**Fig. 2:**
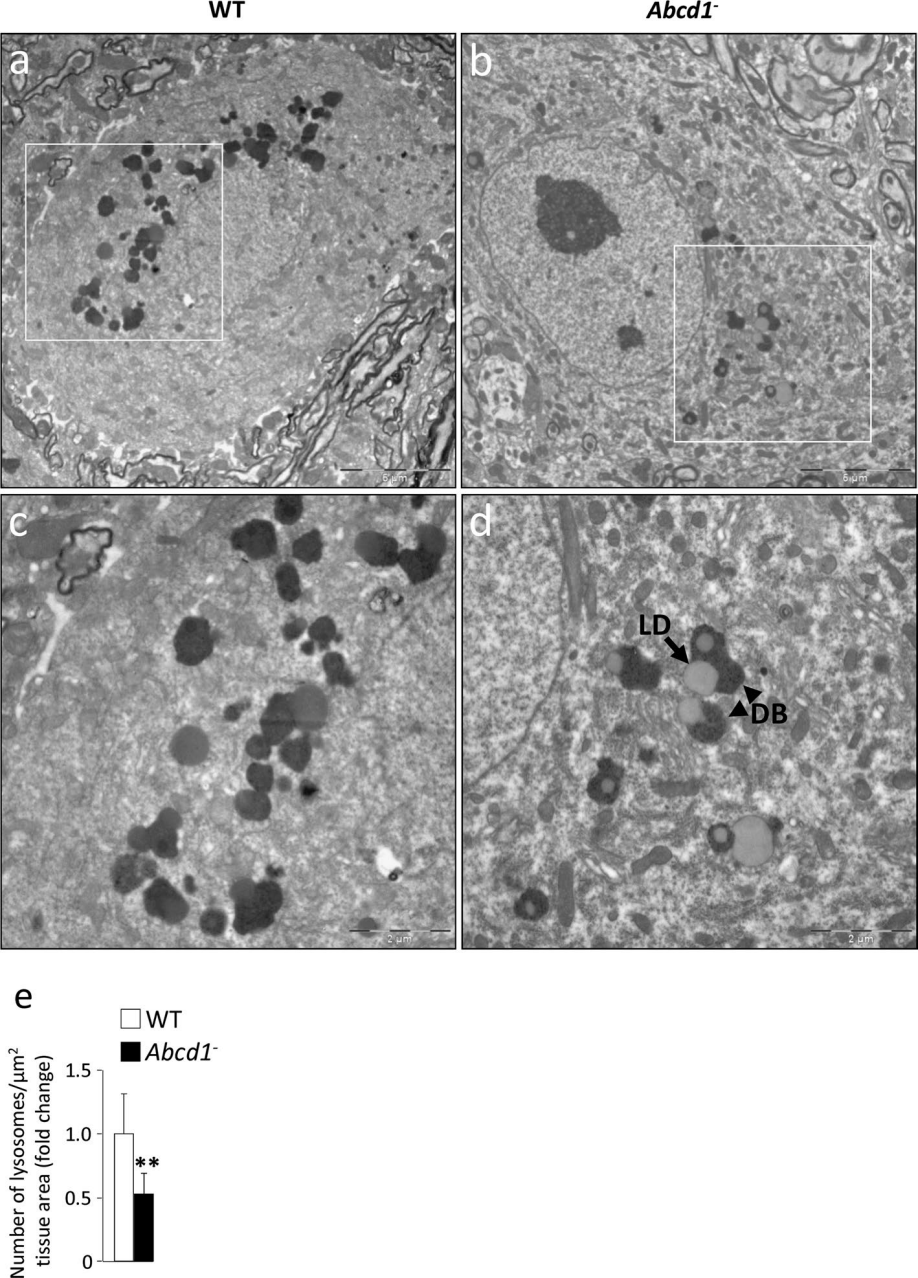
The number of lysosomes decreases in spinal cords of *Abcd1* ^−^ mice. Representative transmission electron microscopic areas of the cell bodies of motor neurons from ventral horns of lumbar sections from spinal cords in 20-month-old WT (**a**, **c**) and *Abcd1* ^−^ (**b**, **d**) mice fixed and stained by conventional procedures as described in “Materials and methods”. The areas shown in **c** and **d** are larger magnifications of the fields marked with a *rectangle* in **a** and **b**, respectively. Notice in **d** the presence of lipid droplets (LD) close to lysosomes of the dense body type (DB). *Bar* 2 µm. **e** The number of dense bodies per µm^2^ motor neuron cell area was counted in more than 30 randomly selected areas from four different mice per genotype. Final counts are shown as mean ± SD relative to WT (**p < 0.01, Student’s *t* test)

Most lysosomes in neurons and other cell types are of the dense body type. They are easily identified by conventional electron microscopy and we observed fewer (by approximately 50 %) motor neuron lysosomes of this type in the *Abcd1* ^−^ mouse spinal cord (as shown in Fig. 2 for 20-month-old mice). Since dense bodies in neurons mainly derive from the cellular autophagic activity, these results are consistent with a decrease in autophagy.

### Autophagic flux is impaired in X-ALD patient fibroblasts

**Fig. 3:**
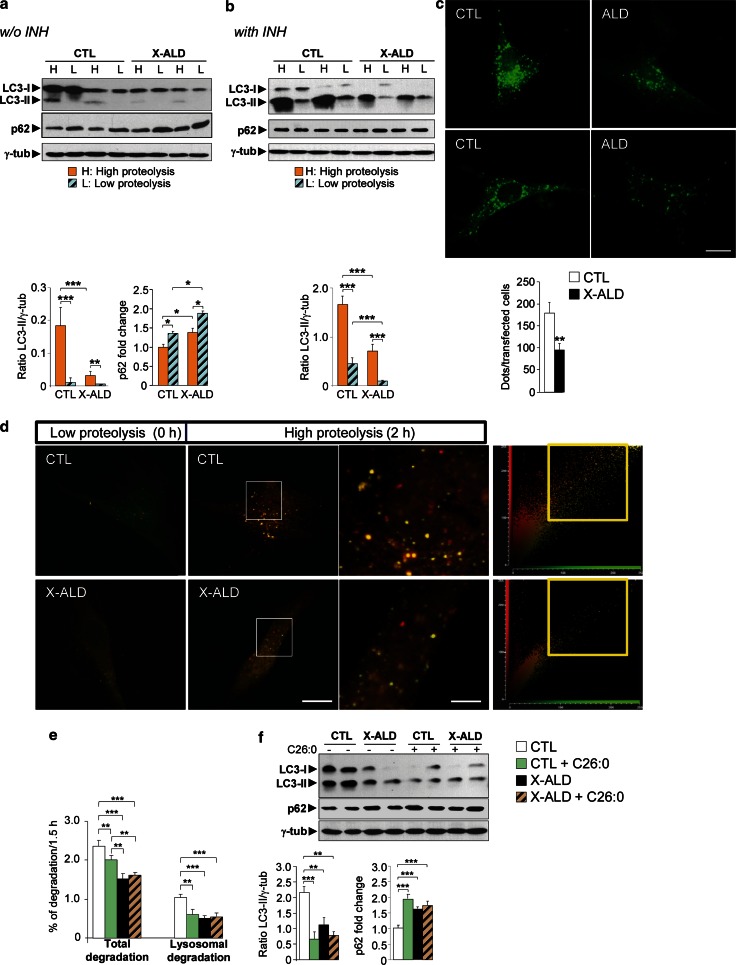
Autophagy is downregulated in fibroblasts from X-ALD patients. **a**, **b** Representative immunoblots for LC3 and p62 in extracts from control (CTL) and patients’ (X-ALD) fibroblasts incubated under high (H) and low (L) proteolysis conditions and incubated without (**a**) or with (**b**) the lysosomal inhibitor bafilomycin A1 (see the “Materials and methods” section for details). The histograms below show the LC3-II levels and the p62 levels relative to the CTL value under high proteolysis conditions. **c** Representative fluorescence images of control (CTL) and X-ALD patients’ (X-ALD) fibroblasts transfected with the EGFP-LC3 plasmid for 48 h and incubated 2 h under high proteolysis conditions. *Bar* 20 µm. The number of fluorescent dots per transfected cell is shown on the histogram *below* (at least 50 transfected cells were counted). **d** Representative fluorescence images of fibroblasts from control (CTL) and X-ALD patients (X-ALD) transfected with the mRFP-GFP-LC3 plasmid for 24 h. The cells were either non incubated (low proteolysis, 0 h) or incubated for 2 h in Krebs–Henseleit medium (high proteolysis, 2 h) as indicated (*two first columns* of images). *Bar* 20 µm. The third column corresponds to higher magnification images of the *square areas* in the images of the *second* *column*. *Bar* 5 µm. The number of both *yellow*, which correspond to autophagosomes, and *red*, which correspond to autolysosomes, puncta are reduced in X-ALD, indicating decreased autophagic flux in these cells. The *fourth column* shows the frequency distribution of intensities in a 2D scatter plot derived from the transfected cells, confirming this conclusion. **e** Control (CTL) and X-ALD fibroblasts (X-ALD) were treated with 50 μM of C26:0 for 24 h. The cells were then labeled with [^3^H]valine in low proteolysis medium and total and lysosomal degradation of long-lived proteins were quantified in high proteolysis medium as described in “Materials and methods”. Results are presented as the percentage of the labeled protein degraded in 1.5 h. **f** Representative immunoblots for p62 and LC3-II in control (CTL) and X-ALD fibroblasts (X-ALD) treated with C26:0 as in **e**. The histograms below show the LC3-II levels and the p62 levels relative to untreated control fibroblasts. Protein levels in **a**, **b** and **f** were always normalized respect to γ-tub. All values are expressed as mean ± SD (*n* = 4 by genotype and condition in **a**, **b**, **e** and **f**; *n* = 3 by genotype in **c**; **p < 0.01 and ***p < 0.001, one-way ANOVA followed by Tukey’s hsd post hoc test for **a**, **b**, **e** and **f**, and Student’s *t* test for **c**)

To monitor autophagy and autophagic flux in X-ALD fibroblasts, cells were cultured as previously described [21] either in a serum- and amino acid-free medium (Krebs–Henseleit medium) to stimulate autophagy via the well-described starvation response (high proteolysis, H) or in complete medium to assess basal autophagy (low proteolysis, L). The fibroblasts from X-ALD patients exhibited lower LC3-II levels and higher p62 levels compared with the controls, especially in H medium (Fig. 3a). Because the observed variations in LC3-II levels (Figs. 1, 3a) can be due to either a change in its synthesis or degradation, human fibroblasts were treated with the lysosomal inhibitor bafilomycin A1 to inhibit LC3-II degradation. Under these conditions, LC3-II levels correlated with the number of autophagosomes in the cells [31]. In the presence of bafilomycin A1, X-ALD patients’ fibroblasts also exhibited lower LC3-II levels compared with control cells in both complete (L) and starvation (H) media (Fig. 3b). As expected, the differences in p62 levels observed in the absence of lysosomal inhibitors in Fig. 3a were normalized in the presence of bafilomycin A1 (Fig. 3b). The decrease in autophagosomes was further confirmed by a lower (approximately 50 % lower compared with the controls) number of fluorescent puncta in X-ALD fibroblasts that transiently expressed EGFP-LC3 (Fig. 3c). Overall, these results support the notion that autophagic flux is impaired in X-ALD fibroblasts. To further reinforce this conclusion, we used an mRFP-GFP-LC3 tandem reporter that has been found useful to trace autophagosome maturation [29] because, in contrast to mRFP that is more stable, GFP is quenched in lysosomes due to the sensitivity of GFP to acid environments. Since in X-ALD fibroblasts yellow fluorescence could be detected, and we found a decrease in both yellow and red fluorescence (Fig. 3d), these results underscore that reduced autophagosome formation and not increased maturation [54] may be responsible for the autophagic defect in these cells.

### VLCFAs impair autophagy in human fibroblasts

In X-ALD, VLCFAs accumulate due to impaired transport and subsequent degradation in peroxisomes [19, 83, 85, 88]. Thus, we sought to determine the putative effect of excess hexacosanoic acid when added to primary fibroblasts isolated from controls and X-ALD patients.

First, we examined whether intracellular protein degradation was altered upon adding excess VLCFAs by analyzing the degradation of long-lived proteins under starvation (high proteolysis) conditions in pulse-chase experiments. We estimated the total level of protein degradation and, separately, lysosomal protein degradation using previously described procedures [21]. As shown in Fig. 3e, the total degradation of long-lived proteins was lower (approximately 35 %) in X-ALD fibroblasts compared with control fibroblasts. Similarly, lysosomal degradation, which mainly corresponds to macroautophagy under these high proteolysis conditions [21], was markedly lower (approximately 50 %). Through alternative methods, these results confirm the impaired X-ALD autophagy detected above and the causal role of excess hexacosanoic acid. Adding excess C26:0 inhibited the total protein and lysosomal degradation levels by approximately 15 and 40 %, respectively, in control fibroblasts; however, no significant additive inhibition was observed in X-ALD fibroblasts (Fig. 3e).

Next, we used immunoblot analyses to determine the LC3-II and p62 levels in cell extracts from control and X-ALD fibroblasts in the presence or absence of excess VLCFAs. In the control fibroblasts, excess VLCFAs induced a marked decrease in the LC3-II levels and an increase in the p62 levels; however, we did not observe a significant difference upon adding excess VLCFAs to X-ALD fibroblasts with previously altered basal LC3-II and p62 levels (Fig. 3f). These results are consistent with the pulse-chase experiments and, thus, suggest a causal effect between the excess VLCFAs and the impaired autophagy in X-ALD patients.

### mTOR activity is enhanced in *Abcd1*^−^ mice

**Fig. 4:**
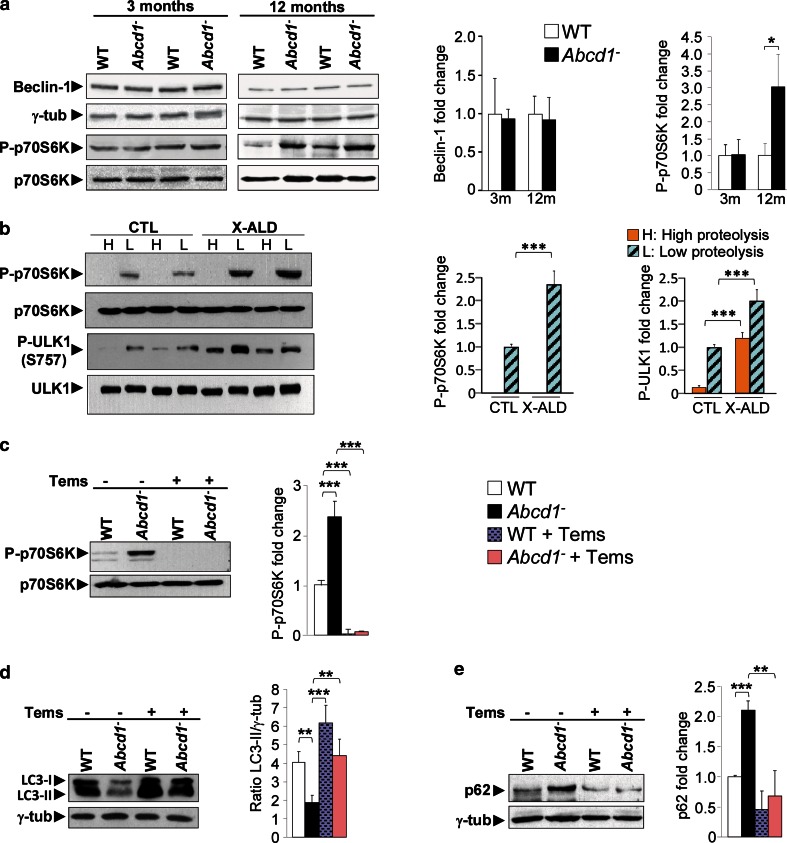
Temsirolimus inhibits mTOR and restores autophagy in spinal cord from *Abcd1* ^−^ mice. **a** Representative immunoblots for Beclin-1 and total p70S6 K and its phosphorylated form (P-p70S6 K) in spinal cords from 3- and 12-month-old WT and *Abcd1* ^−^ mice. The histograms on the *right* show the Beclin-1 levels normalized respect to γ-tub and the P-p70S6 K/p70S6 K ratios relative to their respective WT values. **b** Representative immunoblots for p70S6 K and ULK1 and their phosphorylated forms (P-p70S6 K and P-ULK1) in extracts from control (CTL) and patients´ (X-ALD) fibroblasts incubated under high (H) and low (L) proteolysis conditions. The histograms on the right show P-p70s6 K/p70S6 K and P-ULK1/ULK1 ratios relative to CTL fibroblasts under low proteolysis conditions. Representative immunoblots for p70S6 K and P-p70S6 K (**c**), LC3-II (**d**) and p62 (**e**) in spinal cords of 14-month-old WT mice untreated (WT) or treated with temsirolimus (WT + Tems) and *Abcd1* ^−^ mice untreated (*Abcd1* ^−^) or treated with temsirolimus (*Abcd1* ^−^ + Tems). In **c** and **d**, the histograms on the *right* show, respectively, P-p70S6 K/p70S6 K ratios relative to WT values and the LC3-II levels normalized respect to γ-tub. In **e**, the histogram on the *right* shows the levels of p62 normalized respect to γ-tub and relative to untreated WT mice. All values are expressed as mean ± SD (*n* = 4 samples per genotype and condition in **b**; *n* = 6 samples per genotype and condition in **a** and **c**–**e**; *p < 0.05, **p < 0.01 and ***p < 0.001, one-way ANOVA followed by Tukey’s hsd post hoc test)

Next, we investigated the mechanism underlying the impaired autophagy during X-ALD progression. The class III PI3 kinase complex includes BECLIN-1 and controls pre-autophagosome generation. Because previous studies have shown that lower BECLIN-1 expression may be associated with reduced autophagic vacuole formation [66, 91], we first analyzed BECLIN-1 levels in the X-ALD mouse model. We did not observe significant differences at 3 or 12 months in *Abcd1* ^−^ mouse spinal cords compared with wild-type mice (Fig. 4a). We also determined the activation state of mTOR, which is a major negative regulator of macroautophagy. This kinase inhibits autophagy under the nutrient-rich conditions of a high-fat diet [61, 71]. We analyzed p70S6 K, a well-known mTOR substrate, and showed increased p70S6 K phosphorylation in the spinal cords of 12-month-old but not of 3-month-old *Abcd1* ^−^ mice compared with control mice (Fig. 4a), which is consistent with the results in Fig. 1c. Similarly, we analyzed p70S6 K and the phosphorylation status of ULK1 at Ser 757 (another downstream target of mTOR) in X-ALD fibroblasts, which showed a significant increase under basal conditions (L medium) compared with control fibroblasts (Fig. 4b). As expected, we did not detect p70S6 K phosphorylation in the fibroblasts under starvation conditions (H medium), but the increase in ULK1 phosphorylation (at Ser 757) detected under basal conditions could be also observed, albeit to a lower extent, under starvation.

Because mTOR inhibits autophagy [71], these results suggest that the autophagy defect observed in X-ALD is mTOR dependent.

### Temsirolimus promotes autophagy in *Abcd1*^−^ mice

Because the mTOR inhibitor rapamycin is a well-known autophagy inducer, we sought to test its potential therapeutic effect on X-ALD pathogenesis in vivo. Since rapamycin has poor water solubility and stability in aqueous solutions, we used the rapamycin ester temsirolimus. This drug has more favorable pharmaceutical properties and only induces mild side effects in humans, and it is undergoing evaluation through phase II and phase III clinical trials for treating certain types of cancer [79, 81] (NCT01026792, NCT00827684). Thus, we treated 10-month-old *Abcd1* ^−^ mice with temsirolimus or a vehicle (control) administered through intraperitoneal injection over a 4.5-month period as described above (see the “Materials and methods” section) [52, 67].

Before assessing the effect of temsirolimus in X-ALD, we confirmed that the mTOR pathway was inhibited in the treated mouse spinal cords. As anticipated, mice treated with temsirolimus exhibited lower levels of phosphorylated p70S6 K, but the total p70S6 K immunoreactivity did not change (Fig. 4c). Autophagy was also assessed through measuring the LC3-II and p62 levels using Western blots, which were, respectively, up- and downregulated by temsirolimus in *Abcd1* ^−^ mouse spinal cords (Fig. 4d, e), as expected from mTOR inhibition.

### Temsirolimus prevents oxidized protein accumulation, energetic failure and proteasome malfunction in *Abcd1*^−^ mice

**Fig. 5:**
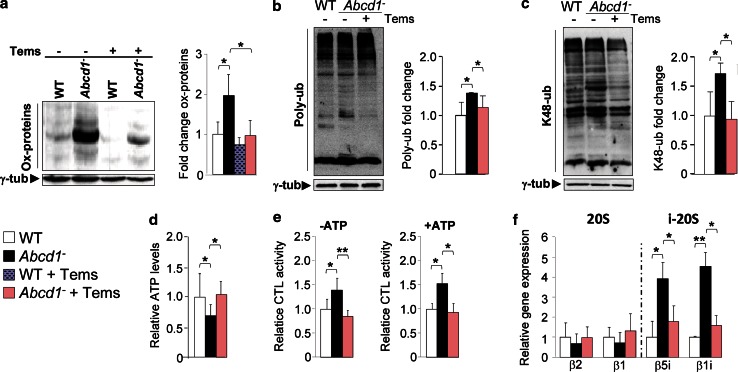
Temsirolimus reduces the accumulation of oxidized proteins, normalizes energetic failure and restores proteasome alterations in spinal cord from *Abcd1* ^−^ mice. Representative immunoblots for oxidized proteins (carbonylated proteins) (**a**), for total (**b**) and for K-48 linked (**c**), poly-ubiquitinated proteins in spinal cords of 14-month-old WT mice untreated (WT) or treated with temsirolimus (WT + Tems) and *Abcd1* ^−^ mice untreated (*Abcd1* ^−^) or treated with temsirolimus (*Abcd1* ^−^ + Tems). In **a**–**c** the histograms on the *right*, show, respectively, the levels of oxidized proteins (**a**), total poly-ubiquitinated (Poly-ub) proteins (**b**) and K-48 linked poly-ubiquitinated proteins (K48-ub) (**c**) relative to untreated WT mice and always normalized respect to the γ-tub. ATP levels (**d**), chymotrypsin-like (CTL) proteasome activities in the presence or not of ATP (**e**) and relative gene expression of 20S (β2, β1) and i-20S (β5i, β1i) proteasome subunits analyzed by quantitative RT-PCR (**f**) expressed relative to untreated WT mice. All values are as mean ± SD (*n* = 4 samples per genotype and condition in (**e**); *n* = 6 samples per genotype and condition in **a**–**c** and **f**; *n* = 8 per genotype and condition in **d**. *p < 0.05, **p < 0.01 and ***p < 0.001, one-way ANOVA followed by Tukey’s hsd post hoc test)

Former studies from our laboratory demonstrated that oxidative stress is a major contributor to X-ALD progression. The data showed signs of oxidative damage in spinal cords from the X-ALD mouse model and in fibroblasts from X-ALD patients with direct oxidative, glycoxidative and lipoxidative damage to proteins, as well as altered enzymatic antioxidant defenses [17]. In addition, we showed that oxidative damage specifically affects ATP levels, which are significantly lower in *Abcd1* ^−^ mice and X-ALD fibroblasts, implying that VLCFA-induced oxidative stress impairs energy metabolism [23]. Although researchers have speculated that autophagy reactivation decreases the oxidative damage propagation in neural tissue, most studies have analyzed whether accumulation of aggregation-prone proteins and cell death decrease without directly examining cellular redox status or oxidative damage [8, 52, 67]. Here, we show that temsirolimus treatment considerably reduced the oxidized protein levels in *Abcd1* ^−^ mouse spinal cords (Fig. 5a) and prevented the associated bioenergetic failure [23], which normalized ATP levels (Fig. 5d).

Finally, our previous studies showed that the ubiquitin–proteasome system (UPS) and immunoproteasome induction malfunctioned as a consequence of and as an adaptive response to oxidative stress during X-ALD pathogenesis [39]. Indeed, the UPS is pivotal in the rapid clearance of damaged, misfolded or aggregated proteins in both healthy and diseased states, and has been shown to play a role in the degradation of oxidized proteins as well [12, 26, 57]. Along with proteasomal degradation, autophagy constitutes the major route for degradation of misfolded or modified intracellular proteins [48]. We therefore hypothesized that induction of autophagy could compensate for both the proteasome and immunoproteasome malfunction by increasing the clearance of oxidized proteins. Thereby, we examined the temsirolimus-mediated effects from these alterations, which showed that the treatment normalized the accumulation of total poly-ubiquitinated proteins (Fig. 5b), and K-48 linked poly-ubiquitinated proteins, which are known to be more specifically targeted for proteasomal degradation (Fig. 5c). The results are, in accordance with the decrease of oxidized proteins (Fig. 5a). Moreover, the treatment also normalized chymotrypsin-like proteasome activity (Fig. 5e) and prevented induction of the immunoproteasome subunits β1i/LMP2 and β5i/LMP7 (Fig. 5f).

### Temsirolimus prevents axonal degeneration in *Abcd1*^−^*/Abcd2*^−*/*−^ mice

*Abcd1* ^−^ */Abcd2* ^−*/*−^ mice exhibit earlier, more severe axonopathy compared with *Abcd1* ^−^ mice, beginning at 12 months of age; therefore, *Abcd1* ^−^ */Abcd2* ^−*/*−^ mice are the preferred model for examining the clinical benefits of a given therapy [44, 50, 56] (see the “Materials and methods” section for details). The double mutants exhibit a neuropathological phenotype characterized by the following: (1) increased labeling with 8-oxo-7,8-dihydro-2′-deoxyguanosine (8-oxodG), which is a marker of oxidative DNA damage, in spinal motor neurons; (2) microgliosis and astrocytosis, as demonstrated by Iba-1 and glial fibrillary acidic protein (GFAP) staining, respectively; (3) axonal damage, as demonstrated by amyloid precursor protein (APP) and synaptophysin accumulation in axonal swellings; and (4) scattered myelin debris, as demonstrated by Sudan black staining [44, 56, 64].

**Fig. 6:**
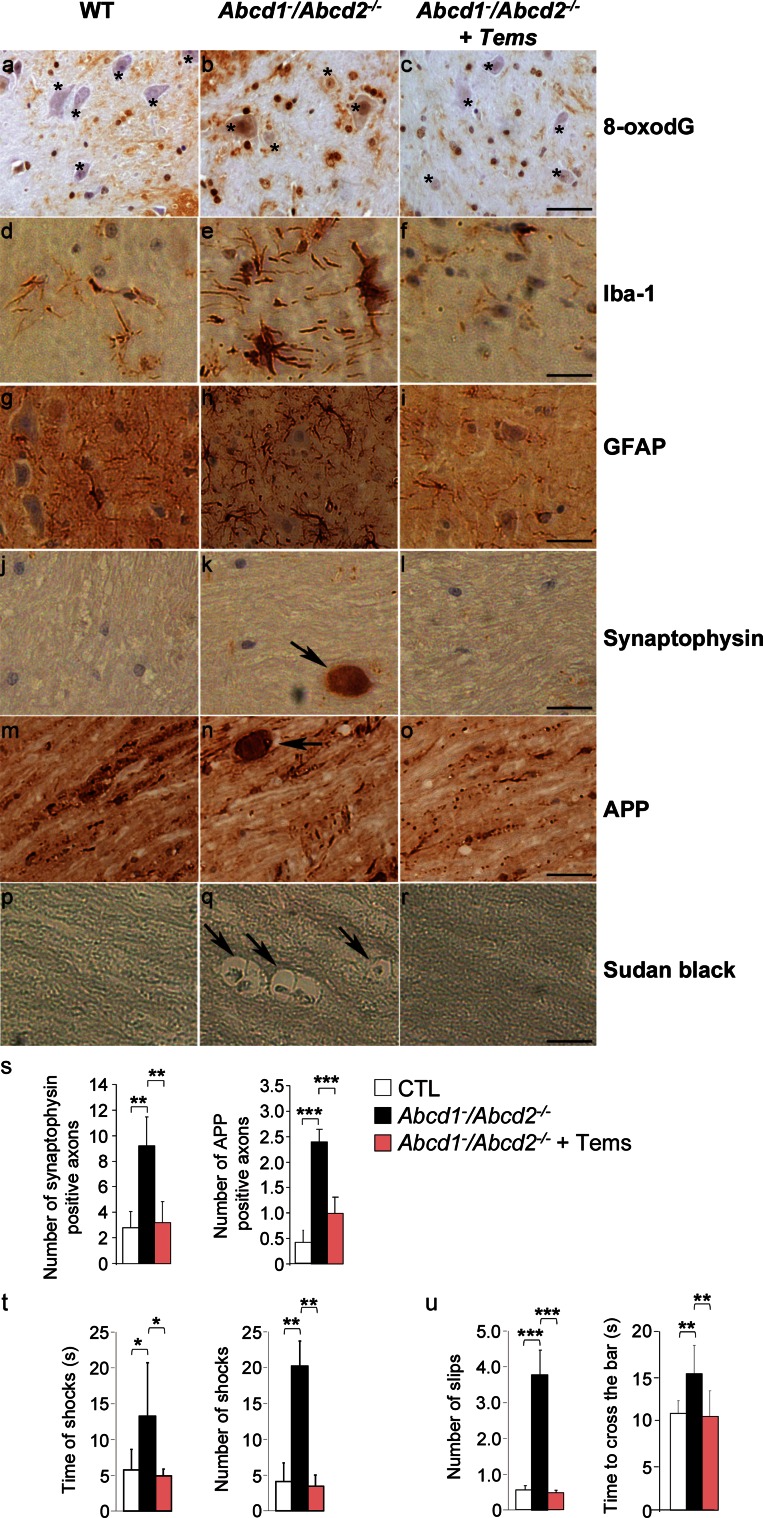
Temsirolimus prevents oxidative stress and myelin and axonal pathologies in spinal cords of 17-month-old *Abcd1* ^−^ */Abcd2* ^−*/*−^ mice. Temsirolimus rescues locomotor deficits in *Abcd1* ^−^ */Abcd2* ^−*/*−^ mice. **a**–**s** Immunohistological analysis of axonal pathologies performed in 17-month-old WT, *Abcd1* ^−^ */Abcd2* ^−*/*−^ and *Abcd1* ^−^ */Abcd2* ^−*/*−^ mice treated with temsirolimus (*Abcd1* ^−^ */Abcd2* ^−*/*−^ + Tems). Spinal cord immunohistological sections were processed for **a**–**c** 8-oxodG, **d**–**f** Iba-1, **g**–**i** GFAP, **j**–**l** synaptophysin, **m**–**o** APP and **p**–**r** Sudan black. Representative images **a**, **d**, **g**, **j**, **m** and **p** for WT, **b**, **e**, **h**, **k**, **n**, and **q** for *Abcd1* ^−^ */Abcd2* ^−*/*−^, and **c**, **f**, **i**, **l**, **o** and **r** for *Abcd1* ^−^ */Abcd2* ^−*/*−^ + Tems mice are shown. *Bars* 25 µm. *Small star* indicates the motor neurons in **a**, **b** and **c**. **s** Quantification of synaptophysin and APP accumulation in spinal cord immunohistological sections of WT, *Abcd1* ^−^ */Abcd2* ^−*/*−^ and *Abcd1* ^−^ */Abcd2* ^−*/*−^ + Tems mice. **t** Treadmill test and **u** bar cross test have been carried out in 17-month-old WT *Abcd1* ^−^ */Abcd2* ^−*/*−^ and *Abcd1* ^−^ */Abcd2* ^−*/*−^ mice treated with temsirolimus (*Abcd1* ^−^ */Abcd2* ^−*/*−^ + Tems). **t** The latency to falling off the belt (time of shocks) and the number of shocks received were computed after 5 min. **u** The time spent to cross the bar and the numbers of slips were quantified. Values are expressed as mean ± SD (*n* = 5 per condition in **a**–**s**; *n* = 12 in **t** and **u**; *p < 0.05, **p < 0.01 and ***p < 0.001, one-way ANOVA followed by Tukey’s hsd post hoc test)

Thus, we treated *Abcd1* ^−^ */Abcd2* ^−*/*−^ mice beginning at disease onset, 12 months of age, for 4.5 months as described (see the “Materials and methods” section). As shown in Fig. 6a–s, treating *Abcd1* ^−^ */Abcd2* ^−*/*−^ mice decreased the accumulation of axonal damage markers as well as the number of reactive astrocytes and reactive microglia to control levels. This treatment also restored the levels of DNA oxidation in spinal motor neurons, which demonstrated cellular specificity for the antioxidant effects found for the whole spinal cords extracts in Fig. 5a.

### Temsirolimus inhibits locomotor deficit progression in A*bcd1*^−^*/Abcd2*^−*/*−^ mice

Locomotor deficits in *Abcd1* ^−^ */Abcd2* ^−*/*−^ mice were evaluated using treadmill and bar cross experiments after temsirolimus treatment. In the treadmill experiment, the double-knockout mice exhibited longer shock times and more shocks compared with wild-type mice, which indicate locomotor disability. Interestingly, temsirolimus treatment normalized these parameters.

In the bar cross experiments, double-knockout mutants often failed to maintain their balance and displayed a greater tendency to slip off the bar as well as longer latencies for reaching the platform at the opposite end of the bar [16, 44, 56]. The number of slips and time necessary to cross the bar were normalized following temsirolimus treatment (Fig. 6t, u). Overall, these data indicate that temsirolimus treatment arrests disease progression in *Abcd1* ^−^ */Abcd2* ^−*/*−^ mice.

## Discussion

**Fig. 7:**
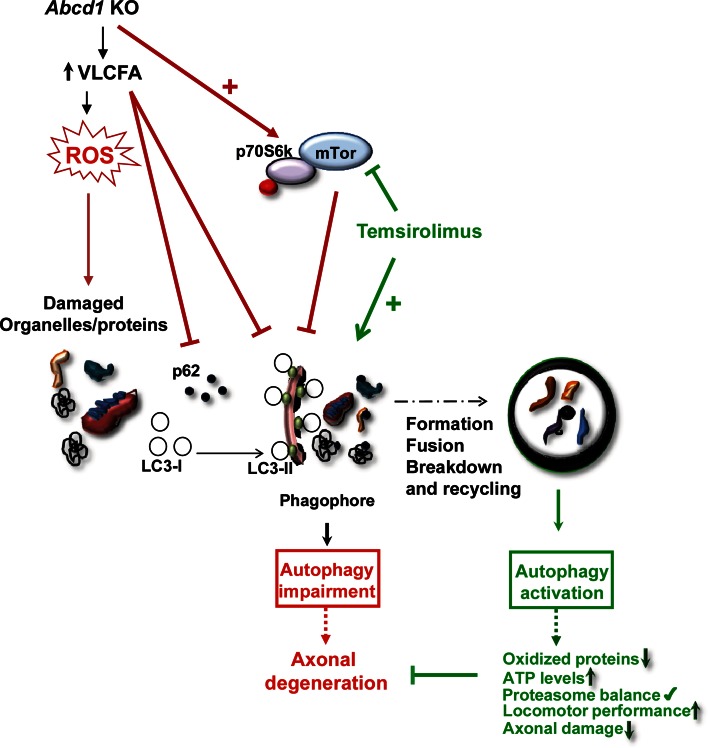
Model illustrating the role of autophagy in the etiopathogenesis of axonal degeneration in X-adrenoleukodystrophy. In X-ALD, VLCFA excess induces production of ROS and accumulation of oxidized proteins and damaged organelles in spinal cord. VLCFA also produce impairment of autophagic flux by inhibition of autophagosome formation. All these alterations (*red lines*) and others result in axonal degeneration. The mTOR inhibitor temsirolimus (*green lines*) restores autophagic flux, prevents oxidized proteins accumulation and bioenergetic failure, leading to protection against axonal degeneration and associated locomotor disability in the *Abcd1* ^−^ */Abcd2* ^−*/*−^ mouse model

Autophagy dysfunction has been directly associated with a growing number of neurodegenerative disorders [1, 48, 58, 89]. Importantly, in previous studies, rapamycin treatment decreased plaques and tangles as well as ameliorated cognitive defects in an AD mouse model, prevented dopaminergic neurodegeneration in parkinsonian mice and reduced the levels of mutant ataxin-3 or mutant huntingtin as well as ameliorated their toxicity in vivo, which confirms previous evidence showing that autophagy induction is beneficial in models of neurodegeneration associated with protein aggregates [11, 47, 52, 67, 71]. Here, we support the notion that impaired autophagy plays a pivotal role in pathogenesis of a neurodegenerative disease unrelated to protein aggregates, X-ALD. In cCALD and cAMN patients, *Abcd1* ^−^ mice and human X-ALD fibroblasts, we show less autophagosome formation as assessed by LC3-II blotting in the presence and absence of a lysosomal inhibitor and other well-established procedures. These experiments could not be performed in human pure AMN samples, due to sample unavailability, although most of the experiments have been carried out in the mouse model for AMN, and the fibroblasts were from AMN patients. Thus, we believe the encountered defects are common to all X-ALD phenotypes, in a similar manner as the accumulation of VLCFA or the oxidative damage is. This decrease of autophagosome formation was associated with an increase in the endogenous autophagy substrate p62 and is likely related to poly-ubiquitinated protein accumulation, which has previously been demonstrated [39]. Impaired autophagy in X-ALD may be due to greater activity by the autophagy negative regulator mTOR. Consistent with these data, we demonstrated that treating the mouse model with temsirolimus, a rapamycin analog that inhibits mTOR, induced autophagy, which enhanced autophagy substrate clearance. In addition, temsirolimus restored proteasome activity, prevented immunoproteasome induction, and, more importantly, inhibited axonopathy progression as well as clinical manifestations. This activity was mediated by normalized signs of oxidative damage and oxidative lesions in protein and DNA as well as the associated energetic homeostasis. Figure 7 summarizes these effects, which indicates that normalizing autophagic function is key for disease progression and, thus, highlights autophagy as a prime therapeutic target. The characteristics seen with the autophagy failure during pathogenesis of X-ALD have both similarities and differences when compared with those found in certain forms of lysosomal storage diseases and other neurodegenerative diseases such as AD and HD. In these cases, autophagic flux was impaired, the number of autophagic vacuoles increased and autophagic substrates accumulated in the affected tissues [49, 89]. However, in Duchenne’s muscular dystrophy, impaired autophagolysosome formation has been observed and is characterized by increased p62 as well as decreased LC3-II and mTOR activation, as we observed in X-ALD. This effect is due to chronic oxidative stress produced by NADPH oxidase in mdx mice [60].

VLCFAs accumulate in X-ALD, and most cells respond to a moderate lipid influx by increasing macroautophagic flow [78]. Similarly, Baarine et al. [3] showed in 2012 that acute VLCFAs exposure could induce autophagy activation, lysosomal membrane destabilization and lysosomal localization changes in wild-type 158 N murine oligodendrocytes. Moreover, upregulated autophagy in response to an acute increase in free saturated fatty acids (e.g., palmitate) was demonstrated in pancreatic beta cell lines (INS-1), neuroblastoma (SK-N-SH), myoblasts (C2C12), and hepatocytes (HepG2) [36]. Furthermore, acute oleic, but not palmitic acid, exposure induces autophagy in HepG2 cells through a mechanism that depends on oxidative stress [51]; however, another group demonstrated that acute exposure to palmitic, but not oleic acid, increases autophagy independent of mTOR in mouse embryonic fibroblasts [80]. Together, these data suggest that free fatty acids regulate autophagy in specific cell types and under particular conditions (acute exposure to free fatty acids). In this study, we provide the first evidence that autophagy is impaired by VLCFAs in control fibroblasts under basal conditions. We did not observe significant changes in X-ALD fibroblasts, which may be explained by the strong inhibition of autophagy already present, most likely due to the increased basal levels of VLCFAs in these cells that may mitigate any exacerbated or adaptive response. Koga et al. [34] observed a reduction in autophagy after acute exposure to a high lipid concentration, or a particular type of lipid or after chronic lipid stimulation (high-fat diet). Mechanism-based studies implicate an inhibitory effect by free fatty acids on autophagy in cells or animals exposed to either high concentrations of free fatty acids (palmitate and oleate) or a prolonged high-fat diet. These observations indicate that there is a primary defect in the fusion of autophagosomes and lysosomes, which is secondary to changes in the lipid composition of those vesicles [34]. These observations are particularly relevant for X-ALD because VLCFAs accumulation has been shown to destabilize and increase the viscosity in model membranes [27]. We recently proposed that excess VLCFAs, either as free fatty acids or part of a lipid complex, interfere with mitochondria membranes and produce ROS through the mitochondrial oxidative phosphorylation system (OXPHOS) [18, 45]. Thus, we posit a similar mechanism by which excess VLCFAs affect lysosomal membranes and inhibit autophagic flux.

Our results highlight autophagy as an efficient, alternative antioxidant defense mechanism that removes oxidized products, not free radicals, as suggested [25]. In addition, a recent study showed that rapamycin directly controls mitochondrial function through promoting aerobic glycolysis over mitochondrial respiration, which reduces oxygen consumption and generates fewer ROS [41]. Furthermore, previous data showed that *TOR1* gene deletion extends the chronological life span in *Saccharomyces cerevisiae* by increasing mitochondrial respiration via enhanced translation of the mtDNA-encoded oxidative phosphorylation complex subunits [7]. Simultaneous restoration of energetic and redox homeostasis through enhanced autophagy induced by temsirolimus is most likely a key factor in arresting disease progression.

Accumulating evidence shows that autophagy stimulation and ROS accumulation are linked to multiple pathological processes, including cancer, neurodegenerative diseases, type-II diabetes, immune diseases and aging [14, 40]. It is tempting to speculate that the impaired autophagy observed in X-ALD may exacerbate oxidative stress and mitochondrial damage, playing a pivotal role in disease progression. Autophagy inhibition produces neurodegeneration in vivo through ubiquitinated protein accumulation, increased ROS and dysfunctional mitochondria [35, 48, 90]. In turn, low mitochondrial ROS levels, which are produced during starvation for example, are necessary for regulating (through posttranslational oxidative modification) Atg4 activity when autophagy activation is part of a survival pathway [73]. These observations are supported by results from wild-type mice subjected to starvation combined with the antioxidant NAC, in which autophagy induction was inhibited, further demonstrating crosstalk between these pathways [82].

Our successful experiments using the rapamycin analog temsirolimus in the *Abcd1* ^−^ and the *Abcd1* ^−^ */Abcd2* ^−*/*−^ mouse model suggest that the therapeutic potential of autophagy induction merits serious consideration. Mice treated with temsirolimus exhibited reduced oxidative damage and improved energetic homeostasis, proteasome activity, neuropathology and scores from two clinical experiments that demonstrate axonopathy. Our results demonstrate a neuroprotective role by temsirolimus in vivo, which suggests a mechanism that attenuates oxidative stress levels. Taken together, our findings strongly suggest that temsirolimus treatment is an attractive therapeutic option for X-adrenomyeloneuropathy patients.

## Electronic supplementary material

Below is the link to the electronic supplementary material.

Supplementary material 1 (DOC 45 kb)
