# Supplementary material for: Autophagy induction halts axonal degeneration in a mouse model of X-adrenoleukodystrophy
Source: Acta Neuropathol. 2014 Dec 31;129(3):399–415. doi: 10.1007/s00401-014-1378-8 (PMC4331612; doi:10.1007/s00401-014-1378-8)
Supplement: Supplementary file 1 — Supplementary material 1 (DOC 45 kb) [file 401_2014_1378_MOESM1_ESM.doc]

**Supplemental Table 1. Human sample description**

| Phenotype | Age | Sex | Cause of death |
| --- | --- | --- | --- |
| CTL | 5 | Male | Accident |
| CTL | 8 | Male | Accident |
| CTL | 12 | Male | Accident |
| CTL | 13 | Male | Accident |
| CTL | 36 | Male | Accident |
| CTL | 46 | Male | Accident |
| CTL | 27 | Male | Accident |
| CTL | 47 | Male | Accident |
| cALD | 6 | Male | X-ALD |
| cALD | 9 | Male | X-ALD |
| cALD | 13 | Male | X-ALD |
| cALD | 13 | Male | X-ALD |
| cAMN | 27 | Male | X-ALD |
| cAMN | 39 | Male | X-ALD |
| cAMN | 39 | Male | X-ALD |
| cAMN | 47 | Male | X-ALD |
